# Idiopathic Atraumatic Renal Hemorrhage: Case Report

**DOI:** 10.5811/cpcem.33528

**Published:** 2025-08-26

**Authors:** Tabitha Ranson, Gregory Ruddy, Zachary Ostapowicz, Leah Joyner

**Affiliations:** * Kansas City University College of Medicine, Department of Emergency Medicine, Joplin, Missouri; † Mercy Hospital, Department of Emergency Medicine, Joplin, Missouri

**Keywords:** spontaneous renal hemorrhage, Wunderlich syndrome, case report, emergent causes of flank pain, atraumatic renal hemorrhage

## Abstract

**Introduction:**

Wunderlich syndrome (WS) is a rare condition characterized by spontaneous, atraumatic renal hemorrhage. It often presents with non-specific symptoms and is typically diagnosed through computed tomography (CT). The most common presentation of WS includes the Lenk triad, which consists of flank pain, a palpable flank mass, and hypovolemic shock. If diagnosis and treatment are delayed, WS can rapidly progress and lead to unfavorable patient outcomes.

**Case Report:**

A 65-year-old male presented to the emergency department with severe sudden-onset left flank pain with subsequent CT angiogram demonstrating an actively bleeding left renal hematoma. The patient was managed conservatively with supportive care. His vitals remained stable, and he did not require any surgical or vascular interventions.

**Conclusion:**

Wunderlich syndrome is a spontaneous renal or perinephric hemorrhage occurring in the absence of trauma; it is rarely included in the differential for patients with flank pain but can become life-threatening when not recognized.

## INTRODUCTION

Atraumatic renal hemorrhage is a condition referred to as Wunderlich syndrome (WS) after it was first described by Carl Wunderlich in 1856.^1^ Acute onset of flank pain, a palpable mass, and hemodynamic compromise is the classic presentation; however, it is relatively uncommon to see all three signs at presentation.^1^–^3^ It is more common for patients to present with unilateral flank pain as the chief complaint and often the only symptom.^3^,^4^ Patients are typically diagnosed with computed tomography (CT) showing subcapsular or perirenal hemorrhage; however, ultrasound and magnetic resonance imaging (MRI) can also be useful in diagnosis.^4^,^5^

Treatment recommendations vary depending on hemodynamics and etiology. Hemodynamically stable patients do well when managed more conservatively, but those exhibiting signs of hemodynamic compromise are usually managed with renal artery embolization.^4^ The majority of spontaneous renal hemorrhage (SRH) cases can be attributed to neoplasms, particularly angiomyolipoma and renal cell carcinoma.^3^,^4^ A smaller proportion of SRH is due to vasculitis or etiologies not otherwise categorized.^4^ Among the other causes contributing to the smallest proportion of cases, etiologies reported include renal artery aneurysms, arteriovenous malformations (AVM), infection, nephrolithiasis, ruptured renal cysts, and uncontrolled hypertension.^4^ Despite the aforementioned causes, occasionally even after thorough investigation there remains a small subset of SRH cases without underlying cause.^4^ The following case describes a 65-year-old male with idiopathic SRH who was successfully treated with conservative measures.

## CASE REPORT

A 65-year-old male presented to the emergency department (ED) with severe sudden-onset left flank pain, reporting that it felt like a prior episode of nephrolithiasis. He denied fever, nausea, vomiting, abdominal pain, dysuria, or hematuria. Past medical history was positive for nephrolithiasis status post lithotripsy, hypertension, hyperlipidemia, type two diabetes mellitus, coronary artery disease status post two-vessel coronary artery bypass surgery, cerebrovascular accident, deep vein thrombosis, pulmonary embolism, and carotid artery stenosis. There was no use of anticoagulants at the time of the ED visit. Social history was positive for former cigarette use (cessation 40 years prior) but negative for alcohol and recreational drug use.

Vital signs on exam were within normal limits, showing blood pressure of 122/55 millimeters of mercury, heart rate 65 beats per minute, and oxygen saturation 100% on room air. Physical exam was significant for moderate to severe distress due to pain, non-tender abdomen, and left costovertebral angle tenderness. Lab work was significant for a hemoglobin of 11.2 grams per deciliter (g/dL) (reference range: 13.5–18.0 g/dL), hematocrit of 36.0% (42.0–52.0%), mean corpuscular volume of 90.9 femtoliters (fL) (78–100 fL), a white blood cell count of 15.0 thousand cells per microliter (k/μL) (4.0–11.0 k/μL), and an acute kidney injury with an elevated creatinine of 1.37 milligrams per dL (mg/dL) (0.67–1.17 mg/dL). Urinalysis was positive for 3+ protein (reference negative), trace ketones (reference negative), and red blood cells (reference negative).

**Image 1:**
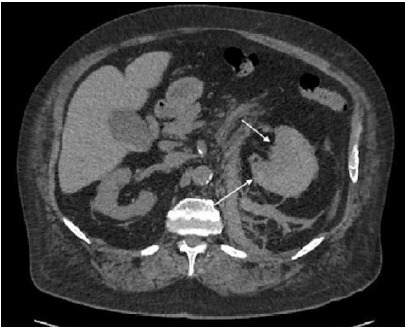
Computed tomography abdomen and pelvis without contrast showing subcapsular renal hematoma of the left kidney (white arrows).

**Image 2:**
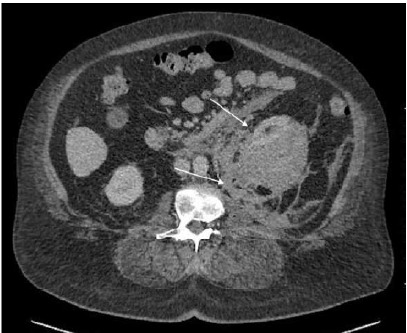
Computed tomography angiogram of the chest, abdomen, and pelvis, axial view, showing subcapsular renal hematoma of the left kidney with active extravasation (white arrows).

Computed tomography without contrast was performed to evaluate for renal calculi and demonstrated an acute left subcapsular perirenal hematoma measuring up to 4.2 centimeters (cm) with surrounding inflammation and hemoperitoneum as well as hemorrhagic right renal cortical cysts (Image 1). Computed tomography angiogram chest, abdomen, and pelvis was performed to further evaluate the bleeding and demonstrated an actively bleeding left renal hematoma measuring 9.4 cm with fat stranding (Image 2).

The patient was admitted to the hospital for further management. During his hospital course, he began to receive packed red blood cells (PRBC) due to a drop in hemoglobin from 11.2 g/dL to 7.0 g/dL, but the transfusion was terminated prior to completion due to an acute febrile transfusion reaction. The patient was monitored with plans for embolization should he become unstable. He was also placed on strict bed rest with strict precautions to avoid any anticoagulant or antiplatelet drugs. His hemoglobin had initially improved from 7.0 g/dL to 8.0 g/dL but then dropped again to 7.3 g/dL on day six of hospitalization, and he required two units of PRBC, which he tolerated after premedication with diphenhydramine and dexamethasone. Ultimately, he required five units of PRBC prior to being discharged. The patient remained hemodynamically stable, and he was discharged home with outpatient urology follow-up.

*CPC-EM Capsule*

What do we already know about this clinical entity?

*Wunderlich syndrome (WS) is a rare condition typically characterized by flank pain, a palpable flank mass, and hypovolemic shock. It is typically diagnosed with computed tomography imaging*.

What makes this presentation of disease reportable?

*Wunderlich syndrome is rare, with few cases seen annually in the emergency department. Even without Lenk triad, it should remain in the differential for atraumatic flank pain to avoid missed diagnosis*.

What is the major learning point?

*This case emphasizes the importance of maintaining a broad differential in cases of vague abdominal symptoms. Point-of-care-ultrasound may be a useful tool in the workup of flank pain*.

How might this improve emergency medicine practice?

*Awareness of WS can improve early recognition, improving patient outcomes. Point-of-care ultrasound in cases of flank pain may improve diagnosis, reduce radiation, and enhance outcomes*.

The patient was seen as an outpatient by urology who were concerned about a possible underlying malignancy in light of the SRH. Repeat imaging four months after initial presentation demonstrated a significant improvement in the left subcapsular renal hematoma now measuring 4.3 x 2.6 cm without any contrast-enhancing masses identified. Additionally, there were several hypodense lesions in the lower pole of the left kidney likely consistent with hemorrhagic cysts. The patient was released from urology follow-up when a CT at seven months post renal hemorrhage was stable, negative for active bleeding, and negative for enhancing mass.

## DISCUSSION

Kidney hemorrhage due to blunt trauma is a rare phenomenon, consisting of only 1–5% of all trauma patients.^6^ Wunderlich syndrome is a clinical syndrome characterized by acute atraumatic renal hemorrhage, which was first reported by Carl Wunderlich in 1856.^1^ Classic presentation of the Lenk triad (flank pain, flank mass, and hypovolemic shock) is seen in only 20% of patients with this type of hemorrhage.^5^ In a case series by Kim et al, the two most common symptoms and signs found in WS are acute-onset flank pain and microscopic hematuria found in 92% and 39% of total cases, respectively.^3^ Although vague symptoms such as flank pain are often associated with more common disorders such as pyelonephritis and nephrolithiasis, WS should remain on the differential particularly in patients with increased risk as it can quickly become life-threatening if not promptly diagnosed and treated. Patients at risk for WS include those with a history of diabetes, hypertension, pyelonephritis, renal cystic disease, and end-stage renal disease.^7^

While several causes of back pain at the costovertebral angle may be treated presumptively without complications including musculoskeletal pain, nephrolithiasis, and uncomplicated pyelonephritis, the management of acute kidney hemorrhage varies greatly. Labs including complete blood count to evaluate for blood loss and infection, basic metabolic panel to assess kidney function, and urinalysis to assess for infection can support the diagnosis and rule out other disorders on the differential. However, an acute kidney hemorrhage can only be accurately diagnosed by imaging.^5^ A CT is currently the gold standard imaging technique in the diagnosis of WS.^8^ If CT is negative and suspicion is still high for WS, then an magnetic resonance imaging may be obtained.^5^

In this specific case, CT without contrast was the initial imaging ordered to rule out renal calculus, particularly with this patient’s history of prior renal calculi. However, the patient then required an additional CT angiogram to further characterize the bleed. Point-of-care ultrasound (POCUS) represents a quick, safe, and cost-effective method to determine possible hemoperitoneum. With the increasing use of POCUS, there have been cases of ultrasound being used to identify and monitor active kidney hemorrhage.^4^,^5^,^9^,^10^ To prevent unnecessary imaging, future standard of care for those with flank pain and risk factors for acute kidney hemorrhage could include POCUS prior to definitive CT to guide choice of imaging modality. If there is evidence of bleeding on POCUS, then CT angiogram can be performed and additional CT can be avoided.

Patients with WS are managed inpatient with renal artery embolization if the patient is unstable, or with supportive care and careful monitoring if the patient remains stable.^4^ The different pathways in managing these patients are important distinctions as renal artery embolization poses potential risk to the patient such as renal failure, hematoma formation at the site of the catheter, arterial hypertension and, lastly, postembolization syndrome.^11^ Thus, if it can be avoided then it is preferred to manage the patient conservatively.^4^

Once diagnosed with an acute renal hemorrhage and stabilized, further investigation typically in the outpatient setting is necessary to rule out secondary causes of WS including malignancy, AVMs, and vasculitides. Thorough examination of the medical history and patient risk factors followed by diagnostic imaging should be performed to evaluate the most likely cause. Exclusion of renal neoplasms via outpatient imaging once the acute bleed and inflammation has had time to resolve is important in the workup of someone with WS, as angiomyolipomas and clear cell renal cell carcinomas contribute to 60–65% of all cases.^5^ If there are no signs, symptoms, or other risk factors for malignancy but no cause has been identified, further workup may be warranted to evaluate for etiologies such as vasculitis or AVMs.^2^

## CONCLUSION

Wunderlich syndrome can present with a wide variety of symptoms that may be misdiagnosed as other common pathologies in the ED. Due to potential for rapid progression of WS and decompensation, it is important to maintain a high level of suspicion when patients with risk factors present with flank pain. Furthermore, when patients present with the classic Lenk triad (left flank pain, hypovolemic shock, and a palpable abdominal mass) interventions should be taken immediately to prevent mortality. While CT is the diagnostic imaging of choice, point-of-care ultrasound at presentation may be a useful adjunct to guide choice of imaging modality. Use of POCUS may assist physicians in ordering the appropriate imaging and decrease unnecessary imaging. Continuing research and clinical education on WS can help emergency physicians identify this disease process and provide patients with earlier interventions leading to a greater number of favorable outcomes.
